# Race/ethnicity and validity of self-reported pneumococcal vaccination

**DOI:** 10.1186/1471-2458-8-227

**Published:** 2008-07-03

**Authors:** Nancy P Gordon, Pascale M Wortley, James A Singleton, Teresa Y Lin, Barbara H Bardenheier

**Affiliations:** 1 Kaiser Permanente Division of Research, Oakland, CA, USA; 2 Immunization Services Division, National Center for Immunizations and Respiratory Diseases, Centers for Disease Control and Prevention, Atlanta, GA, USA

## Abstract

**Background:**

National and state surveys show large disparities in pneumococcal vaccination status among Whites, Blacks and Latinos aged ≥ 65. The purpose of this study is to determine whether there is any difference in the validity of self-report for pneumococcal vaccination by race/ethnicity that might contribute to the substantial disparities observed in population-level coverage estimates.

**Methods:**

Self-reported vaccination status was compared with medical record documentation for samples of White, Black, and Latino members of a large health plan to examine whether differences in validity of self-report contribute to observed disparities.

**Results:**

Sensitivity was significantly lower for Blacks (0.849, 95% CI 0.818–0.876) and Latinos (0.869, 95% CI 0.847–0.889) than for Whites (0.931 95% CI 0.918–0.942). Specificity was somewhat higher for Blacks than for Latinos and Whites, but the differences were not statistically significant. Coverage for Whites, Blacks and Latinos, respectively, was 84.3%, 73.5%, and 82.3% based on self-report, but 74.8%, 71.9%, and 84.2% based on medical records.

**Conclusion:**

The results of this study suggest that differential self-report error, i.e., summative effect of over-reporting and under-reporting within a race-ethnic group, may contribute to the size and direction of race-ethnic disparities in pneumococcal vaccination observed in surveys.

## Background

Invasive pneumococcal disease accounted for approximately 3,400 deaths per year among persons aged 65 and over during 1990–1999 [1]. CDC's Advisory Committee on Immunization Practices (ACIP) recommends a single dose of pneumococcal polysaccharide vaccine for all people 65 years and older [2]. Healthy People 2010 Objectives call for ≥ 90% of adults aged ≥ 65 to have had a pneumococcal vaccination [3], and pneumococcal vaccination of adults in this age group has been made a performance measure for the 2004 Health Plan Employer Data and Information Set (HEDIS) sponsored by the National Committee for Quality Assurance (NCQA). National surveys have found that while pneumococcal vaccination coverage has significantly increased over the years, vaccination rates of Black and Hispanic/Latino seniors are 17 to 30 percentage points lower than for Whites [4-6]. These differences persist even among seniors with higher likelihood of receiving preventive services, i.e., those with health insurance [5,6] and with at least some college education [7]. The reasons for the disparities are yet to be well understood and are probably multifactorial [8,9].

Estimates of pneumococcal vaccination coverage are based on self-reported information from state (e.g., Behavioral Risk Factor Surveillance System (BRFSS)) and national (e.g., National Health Interview Survey (NHIS)) surveys, or at a more local level, from health plan member surveys conducted for quality of care reporting purposes. Recall for pneumococcal vaccination has been shown to be less accurate than for influenza vaccination [10-12] presumably at least in part because the event may have occurred in the more distant past. In addition, because awareness about the vaccine is less [8], recall of its receipt may thus be less. The purpose of this study is to determine whether there is any difference between Blacks, Hispanics, and Whites in the validity of self-report for pneumococcal vaccination that might contribute to the substantial disparities observed in population-level coverage estimates. The Kaiser Permanente Medical Program in Northern California offered the opportunity to conduct a study where self-report of vaccination could be compared to medical records in a diverse population of over 380,000 members aged 65 and over. This survey and its use to conduct this validation study was approved by the Kaiser Foundation Research Institute Institutional Review Board.

## Methods

### Source of self-reported vaccination status

Every three years since 1993, Kaiser Permanente in Northern California has conducted an adult Member Health Survey, a self-administered mailed survey which covers demographic characteristics, health status, health conditions, behavioral health risks, and receipt of preventive and patient education services. The overall response rate among adults aged ≥ 65 was 74% for both the 1999 and 2002 surveys, yielding approximately 7,100 respondents each year. Combining 1999 and 2002 respondents yielded 11,182 men and women aged 66–85 who self-identified as White/Euro-American (White), African American/Black (Black), or Hispanic/Latino (Latino). Respondents were asked if they ever had a pneumonia shot, with answer options of "Yes, at Kaiser," "Yes, but not at Kaiser," "No," or "Don't Know/Don't remember." Estimates of pneumococcal vaccination coverage based on data from the 2002 survey weighted to the adult membership at the time of the survey are shown in Table 1.

**Table 1:** Estimated Percentages of White, Black, and Latino Health Plan Members Aged 66–85 Who Ever Had a Pneumococcal Vaccination, 2002^a^

**Group**	**% Pneumococcal Vaccination, Excluding Don't Know and Missing Data from Denominator**	**% Pneumococcal Vaccination, Including Don't Know and Missing Data in Denominator**	**% with Unknown Pneumococcal Vaccination Status**
	% (95% CI) (Sample N)	% (95% CI) (Sample N)	% (95% CI)
ALL			
White	83.7% (82.5% – 85.0%) (N = 4573)	77.6% (76.2% – 78.9%) (N = 4945)	7.4% (6.5% – 8.2%)
Black	78.2% (72.6% – 83.8%) (N= 273)	67.8% (61.8% – 73.7%) (N = 316)	13.3% (9.0% – 17.7%)
Latino	78.5% (73.5% – 83.5%) (N = 351)	69.5% (64.3% – 74.7%) (N = 400)	11.5% (8.0% – 15.0%)

FEMALES			
White	85.0% (83.3% – 86.7%) (N = 2337)	81.0% (79.2% – 82.8%) (N = 2451)	4.7% (3.7% – 5.7%)
Black	76.5% (68.8% – 84.1%) (N = 149)	66.7% (58.8%–74.7%) (N = 171)	12.7% (7.1% – 18.3%)
Latina	79.9% (73.2% – 86.6%) (N = 185)	73.7% (66.8% – 80.6%) (N = 202)	7.8% (3.9% – 11.7%)

MALES			
White	82.1% (80.2% – 84.0%) (N = 2236)	73.5% (71.4% – 75.5%) (N = 2494)	10.5% (9.1% – 11.9%)
Black	80.6% (72.5% – 88.7%) (N = 124)	69.2% (60.2% – 78.2%) (N = 145)	14.2% (7.2% – 21.1%)
Latino	76.4% (69.0% – 83.8%) (N = 166)	63.8% (56.0% – 71.5%) (N = 198)	16.6% (10.4% – 22.8%)

^a ^Based on self-report data from the 2002 Kaiser Permanente Adult Member Health Survey weighted to reflect the age, gender, and geographic composition of the membership at the time of the survey.

### Study population

Subjects for this study were selected from a pool of current and former health plan members aged 66–85 who had responded to either the 1999 or 2002 Kaiser Permanente Member Health Survey, were able to comprehend English (since the questionnaires were only available in English), self-identified as White, Black, or Latino (but not more than one race-ethnicity) on the questionnaire, and had been continuous members of the Health Plan from within 3 months after turning age 65 through the date the survey questionnaire was returned. From this eligible pool of survey respondents, for each of the 3 race-ethnic groups, samples of up to 100 men and 100 women who had indicated receiving a pneumococcal vaccination (hereafter, pneumovax) from the Health Plan (i.e., not from another source), and up to 100 men and 100 women who had indicated never having had this vaccination were selected. The final study sample consisted of 400 nonHispanic Whites (hereafter referred to as Whites), 300 nonHispanic Blacks (hereafter referred to as Blacks), and 261 Hispanics/Latinos (hereafter referred to as Latinos), most of whom were of Mexican or Central American descent). All eligible Blacks and Latinos were included, but Whites were randomly sampled as follows: Pneumovax Yes: 100/2419 men and 100/2724 women; Pneumovax No: 100/472 men 100/472 and 100/486 women.

### Health plan medical record documentation of vaccination status

Health Plan medical records for each of the 961 individuals in the final sample were searched for a retrospective interval of up to 10 years prior to the age of 65 (depending on the age at which the individual had joined the health plan) through the date the self-reported information was received to determine whether there was any record that the individual had received a pneumovax from the Health Plan or from outside the Health Plan (as reported in a chart note). First searched was the Health Plan's immunization tracking database (KITS), which by January 1995 captured the dates of all health plan-administered pneumococcal vaccinations. Subjects with a pneumovax date in the immunization tracking database that came after the date the questionnaire was completed were assumed to be unvaccinated prior to the provision of the self-report. For the 137 out of 961 study subjects without a pneumovax date in the immunization tracking database, comprehensive reviews of all hardcopy medical charts from every outpatient clinic and hospital where the individual received care during their retrospective study interval were completed, irrespective of whether they had reported a pneumovax or not.

### Data analysis

The validity of self-report compared with Health Plan medical records (HPMR) was assessed for each race/ethnic group by calculating rates of sensitivity, specificity, false positives, and false negatives, assuming the HPMR to be the "gold standard" of accuracy. Sensitivity ("true positives") is the proportion of those with a pneumovax documented in the HPMR that reported having had a pneumovax. Specificity ("true negatives") is the proportion of those with no pneumovax documented in the HPMR who reported never having had a pneumovax. The proportions under- and over-reporting vaccination correspond to 1-sensitivity and 1-specificity, respectively. Positive Predictive Value (PPV), the complement of the false positive rate, is the proportion of those who reported a pneumovax who had this confirmed by the HPMR. Negative Predictive Value (NPV), the complement of the false negative value (FNV), represents the proportion of those who reported no pneumovax who had this confirmed by the HPMR. These analyses were conducted using SUDAAN [13]. Kappa statistics were also calculated to determine the degree of agreement. Strength of agreement is considered almost perfect for kappa values ≥ 0.81; very good for values of .61 to .80; moderate for values of .41 to .60; fair for values of .21 to .40; and poor for values =.20 [14].

Logistic regression models were used to test whether sensitivity and specificity differed by race/ethnicity after controlling for (1) age and gender and (2) age, gender and education, and (3) age and education in separate models for men and women. The modeling of sensitivity was restricted to people whose HPMR documented receipt of a pneumovax, and the modeling of specificity was restricted to people with no HPMR documentation of a pneumovax. The covariates were represented by sets of indicator variables: gender, female vs. male; age, 70–74, 75–79, 80–85 vs. 66–74; and education, < 12^th ^grade, some post-high school, college graduate vs. high school graduate or equivalent. The logistic regression procedure in SAS was used to conduct these analyses [15].

To correct for the artificial distribution of vaccinated and unvaccinated persons within each of the six Race-Ethnicity (nonHispanic White, nonHispanic Black, Hispanic/Latino) × Gender study groups created by the sampling design, all study data were weighted to reflect the actual distribution of self-reported pneumovax status among study eligibles within each of the groups (see Appendix for description of how weighting factors were created). All statistical analyses used these weighted data.

## Results

Characteristics of the final White, Black, and Latino study groups are shown in Table 2. The Black group was fairly similar to the White group with respect to age and education, while the Latino group was younger and less educated than the White group. Among those with a documented pneumovax, the length of time between documented pneumovax date and self-report date was fairly consistently distributed across all three race-ethnic groups, with approximately 1/3 having an interval of ≤ 2 years and another 1/3 having an interval of > 2 to 5 years. However, 12% of the White and Latino male groups had an interval of greater than 7 years.

**Table 2:** Selected Characteristics of Study Subgroups ^a^

	**White**			**Black**			**Latino**		
			
**Characteristics**	**All** (N = 400) **Wtd %**	**Women** (N= 200) **Wtd %**	**Men** (N = 200) **Wtd %**	**All** (N = 300) **Wtd %**	**Women** (N = 156) **Wtd %**	**Men** (N = 144) **Wtd %**	**All** (N = 261) **Wtd %**	**Women** (N = 131) **Wtd %**	**Men** (N = 130) **Wtd %**
Age									
66–69	18.9	19.4	18.4	22.5	24.5	20.0	25.7	32.3	17.8
70–74	29.0	30.2	27.7	31.4	29.1	34.2	31.0	30.4	31.8
75–79	44.1	43.9	44.2	36.9	37.9	35.6	35.6	30.5	41.7
80–85	8.0	6.5	9.7	9.3	8.5	10.2	7.7	6.9	8.7

Education									
< 12 years	13.5	12.5	14.5	23.4	21.5	25.6	35.7	34.0	37.7.
High School Grad	27.1	34.7	18.7	24.6	26.9	21.7	23.8	30.8	15.4
Some College	37.0	32.6	41.9	36.5	36.8	36.3	25.5	24.0	27.3
College Grad	22.4	20.3	24.8	15.6	14.8	16.4	15.0	11.3	19.5

High Risk ^b ^(Yes)	42.1	32.6	52.8	53.1	47.4	60.1	46.1	40.1	53.4.

Self-reported Pneumococcal Vaccination (Yes)	84.3	84.9	83.7	73.5	73.0	74.1	82.4	83.7	80.9

HPMR documented Pneumococcal Vaccination (Yes) ^c^	74.8	76.1	73.5	72.1	73.9	69.9	84.0	82.0	86.5.

Interval between HPMR date and Self-Report date ^d^									
<= 2 yr	34.5	34.8	34.1	33.9	31.6	36.9	35.0	34.9	35.2
> 2–3 yr	12.4	12.9	11.8	17.1	19.4	14.2	14.7	14.2	15.2
> 3–5 yr	29.8	30.7	28.6	28.4	28.0	29.0	27.2	33.1	20.4
> 5–7 yr	14.7	15.8	13.4	13.2	13.5	12.9	13.2	10.1	16.8
> 7–10 yr	7.4	4.7	10.4	5.4	4.9	5.9	8.5	6.8	10.5
> 10 yr	1.3	1.1	1.6	1.9	2.6	1.1	1.4	1.0	1.9

^a ^Results are based on data weighted to reflect actual distribution of self-reported pneumonia vaccination status among all eligible study subjects in each race-ethnicity × gender group.

^b ^High Risk = Reported one or more of the following health conditions: diabetes, heart problem, lung/breathing problem (e.g., asthma, COPD), or cancer.

^c ^Pneumococcal vaccination date found in Health Plan Medical Record (HPMR) or written note in patient chart about date individual had the vaccination outside the Health Plan.

^d ^For people with HPMR documentation of pneumococcal vaccination only.

The proportion of subjects with documented vaccination who reported vaccination (sensitivity, or "true positives"), was significantly lower for Blacks and Latinos than for Whites; correspondingly, under-reporting of vaccination (1-sensitivity) was more common among Blacks and Latinos than Whites. The proportion of subjects without documentation of vaccination who reported being unvaccinated (specificity or "true negatives") was low, ranging from 0.415 to 0.56. This finding indicates that over-reporting (1-specificity) was common. Specificity was higher for Blacks compared with Latinos and Whites, but these differences were not statistically significant. PPV, the proportion of those reporting vaccination who have documentation of vaccination, did not vary across groups, but NPV, the proportion of those reporting being unvaccinated who have no chart documentation of vaccination, was lower for Blacks and Latinos than Whites, with the latter difference being statistically significant (Table 3). The kappa statistics ranged from .28 for Latinos (fair agreement) to .40 and .42 (just barely moderate agreement) for Whites and Blacks, respectively (Table 3).

**Table 3:** Measures of Validity and Agreement between Self-Report and Health Plan Medical Records, by Race/Ethnicity^a^

**Group**							**Validity of Self-Reported Measures of Pneumococcal Vaccination**				**Measures of Agreement**	
			
	**Unwtd Ns.**			**Wtd. Ns**								
			
	**SR**	**HPMR**		**SR**	**HPMR**		**Sensitivity** **(95% CI)**	**Specificity** **(95% CI)**	**PPV** **(95% CI)**	**NPV** **(95% CI)**	**Kappa**	**Concordance**
										
		Yes	No		Yes	No						
White	Yes	165	35	Yes	278	59	.931	.419	.826	.671	.395	80.0%
(n = 400)	No	66	134	No	21	42	(.918–.942)	(.349–.491)	(.773–.870)	(.609–.728)	(.293–.485)	
Black	Yes	166	34	Yes	183	37	.849	.560	.833	.590	.413	76.7%
(n = 300)	No	41	59	No	33	47	(.818–.876)	(.479–.637)	(.779–.875)	(.498–.676)	(.296–.520)	
Latino	Yes	178	22	Yes	191	24	.869	.415	.887	.377	.277	79.8%
(n = 261)	No	38	23	No	29	17	(.847–.889)	(.307–.532)	(.838–.923)	(.272–.495)	(.133–.419)	

^a ^Results are based on data weighted to reflect actual distribution of self-reported pneumonia vaccination status among all eligible study subjects in each race-ethnicity × gender group.

SR = Self-report from questionnaire. HPMR = Health Plan medical record documentation of a pneumonia vaccination prior to the date of the self-report.

Unwtd Ns show 2 × 2 distributions pre-weighting; Wtd N's, rounded to integer form, show the 2 × 2 distributions after post-stratification weighting.

The logistic models revealed no race/ethnic differences for specificity, but sensitivity was significantly lower among Blacks and Latinos compared with Whites in models controlling for gender and age and gender, age and education (Black vs. White: OR = .41, CI: .23–.74; Latino vs. White: OR = .48, CI: .27–.88, with no appreciable effect of education). Gender-specific analyses found these differences to be statistically significant only among the women.

Comparing estimates based on self-report to Health Plan medical record data, coverage based on self-report was substantially higher than coverage based on health plan records for the White group (84.3% vs. 74.8%), while the estimates for the Black and Latino groups were more concordant (73.5% vs. 72.1% and 82.4% vs. 84.0%, respectively).

## Discussion

This study compared self-report and medical records for a sample of Black, Latino, and White seniors to examine whether there are race-ethnic differences in validity of self-reported pneumococcal vaccination status, and if so, to explore whether this source of error could help to explain the persistent race-ethnic disparities in pneumococcal vaccination rates observed in national health surveys. We found that in this sample of Kaiser Permanente health plan members who met study eligibility requirements, the Black-White gap in vaccination was substantially less based on chart documented vaccination compared with self report. Because this is a highly vaccinated population in a health care system that stresses the importance of vaccination and other clinical preventive services, the estimates of under-reporting (lack of sensitivity) and over-reporting (lack of specificity) from this study cannot be directly applied to other populations. Nonetheless, the findings suggest that differential validity of self-report may contribute to disparities observed in national self-reported data.

The balance between under- and over-reporting ultimately determines to what extent true and reported coverage differ. In our sample, over-reporting of vaccination was high in all 3 groups, but vaccinated Blacks and Latinos were less likely to report vaccination than vaccinated Whites. For Blacks and Latinos, coverage by self-report and record validation were similar because under- and over-reporting balanced out. For Whites, however, self-reported coverage was higher because a lower rate of under-reporting resulted in inflated rates. The higher rate of under-reporting of vaccination by Blacks and Latinos could in part be related to differences in awareness of the vaccine across groups. For example, in a recent study, elderly Blacks and Latinos were less likely than elderly Whites to know that pneumococcal vaccination is recommended for persons their age [8] and in another, elderly Blacks, but not Latinos, were less likely to be aware of the recommendation [16]. Potential differences in doctor-patient communication also could account for some of the observed difference [17]. Over-reporting, on the other hand, could result from confusion with influenza vaccine or an assumption that pneumococcal vaccination had been received.

Previous studies of validity of self-report found that overall sensitivity of self-report (accuracy of recall about having been vaccinated) is generally higher than specificity (accuracy of recall about not having been vaccinated) [10-12]. Sensitivity has ranged from 0.75 to 0.97, while specificity has ranged from 0.25 to 0.83. The greater variation in specificity may largely be a reflection of the variation in quality of the medical records or in the ability to review the relevant records. In addition, populations studied varied and may have differed in their propensity to under- or over-report. It must be noted that race/ethnicity-specific findings from our study cannot be applied to other populations as sensitivity and specificity may differ in different populations, and could even differ in the same population over time.

Major strengths of this study include the ability to assess validity of self-reported vaccination by race/ethnicity and the thoroughness of the medical record review compared to previous studies. Measures were taken to improve the ascertainment of pneumococcal vaccination, i.e. restricting the study to persons who had been continuous members of the health plan from at least age 65 through the date that the self-report was collected, and searching all of the individual's health plan medical records for information about vaccination status as far as 10 years before the person turned 65. However, it is unclear how well medical record documentation serves as a "gold standard" for vaccination status. While it can probably be assumed that documented history of pneumococcal vaccination indicates that the person really was vaccinated, lack of documentation may not reflect true vaccination status. Nevertheless, medical record review, while imperfect, has been the method used to validate self-report in previous studies [10-12]. It may be more accurate to say that among those persons with documented vaccination, self-report of vaccination differed significantly by race/ethnicity, and while the findings cannot definitively assess the validity of self report, these differences do suggest that differences in the validity of self-report by race/ethnicity may exist. Other limitations include the sample size and the fact that the study subjects were all members of one health plan and not representative of the U.S. population. In addition, the Latinos in the sample were primarily from Mexico and Central America and needed to be able to complete a questionnaire in English. Thus, the results for this ethnic group may not be generalizable to ethnic Hispanics from Puerto Rico, Cuba, the Caribbean, and other parts of the Spanish-speaking world, nor to people who cannot read English.

Self-report is relied on for monitoring progress towards Healthy People 2010 goals, as well as for HEDIS measures. Self-reported coverage can be substantially biased or can be similar to true coverage, depending on the balance between under and over-reporting. In this study, an apparent disparity between Blacks and Whites in self-reported coverage resulted from differences in recall, while an actual disparity between Whites and Latinos was obscured by differences in recall. These findings raise the question whether record-based approaches should be considered in the health plan setting. However, since many health systems do not however have systems like the Kaiser immunization registry, this could be a substantial challenge. In this study, reports of persons who responded "don't know" were not validated. In the Kaiser Permanente Member Health Survey, the proportion of seniors who did not know their pneumococcal vaccination status was larger than in national surveys such as the NHIS or BRFSS, possibly as a result of lack of prompting in a mail survey compared with in-person or telephone surveys, and Black and Latino seniors were more likely to fall into this category than White seniors. Validation studies should be conducted to better understand how those who respond "don't know" differ, and multiple imputation methods should be used to quantify potential bias when a substantial portion of respondents report "don't know". National and state-level trends are monitored through self-report in population-based surveys like the National Health Interview Survey and the Behavioral Risk Factor Surveillance System. Record-based approaches are not practical for such surveys. Further studies should, however, be conducted in population-based settings to measure validity of self-report across racial/ethnic groups and within groups over time.

## Conclusion

The results of this study suggest that differential self-report error, i.e., summative effect of over-reporting and under-reporting within a race-ethnic group, may contribute to the size and direction of race-ethnic disparities in pneumococcal vaccination observed in surveys.

## Appendix

### How study weighting factors were created

To correct for the artificial distribution of vaccinated and unvaccinated persons, all study data were weighted to reflect the actual distribution of self-reported pneumovax status among study eligibles in each of 6 Race/Ethnicity × Gender study samples. Specifically, for each of 12 study subgroups (3 Race/Ethnicity × 2 Gender × 2 Self-Reported Pneumovax Status), weighting factors were created by dividing the number of study eligibles from that subgroup by the final number selected for the study sample. The 12 subgroups were then collapsed into 6 Race/Ethnicity × Gender groups and the weighted Ns for the two self-reported pneumovax strata were summed to create a group weighted N. The resulting 6 samples, after weighting, reflected the underlying distribution of self-reported pneumovax status among all study eligibles. Finally, we created a "deflated" weighting factor which resulted in the sum of weighted Ns in each of the 6 groups equaling the total sample Ns for these groups, while keeping the same distribution of self-reported pneumovax status as the undeflated weighting factor. The "deflated" weighting factors were used for all analyses.

## Competing interests

The authors declare that they have no competing interests.

## Authors' contributions

PMW conceived the study, collaborated on the study design, the structuring of the statistical analysis, interpretation of the data, and writing of the manuscript. NPG collaborated on the design of the study, was responsible for overall conduct of the study, collaborated on the analysis and interpretation of the data, and took the lead in drafting the manuscript. TYL collaborated on the design of the study, was responsible for data collection and data management, performed preliminary data analysis, and participated in the writing of the manuscript. JAS assisted with the structuring of the statistical analysis and interpretation of the data and participated in the writing of the manuscript. BHB assisted with the data analysis and participated in the writing of the manuscript.

## Pre-publication history

The pre-publication history for this paper can be accessed here:


